# Exploring social determinants of health in a Saudi Arabian primary health care setting: the need for a multidisciplinary approach

**DOI:** 10.1186/s12939-022-01627-2

**Published:** 2022-02-16

**Authors:** Basmah Almujadidi, Alayne Adams, Aljohara Alquaiz, Gerald Van Gurp, Tibor Schuster, Anne Andermann

**Affiliations:** 1 Department of Family Medicine, McGill University, Montreal, Quebec Canada; 2 Department of Family & Community Medicine, King Saud University, Riyadh, Kingdom of Saudi Arabia; 3 Present Address: St Mary’s Research Centre, McGill University, Montreal, Quebec Canada

**Keywords:** Frontline health workers, Primary healthcare physicians, Social determinants of health, Underserved patients, Social accountability, Qualitative research

## Abstract

**Background:**

Action on social determinants of health (SDH) in primary health care settings is constrained by practitioners, organizational, and contextual factors. The aim of this study is to identify barriers and enablers for addressing SDH in clinical settings in Saudi Arabia, taking into consideration the influence of local cultural and social norms, to improve care and support for marginalized and underserved patients.

**Methods:**

We conducted a qualitative study involving individual in-depth interviews with a sample of 17 primary health care physicians purposefully selected based on the inclusion criteria, as well as a focus group with four social workers, all recruited from King Khalid University Hospital (KKUH) in Riyadh, Saudi Arabia. All interviews were audio-recorded, translated from Arabic to English, transcribed verbatim, and analyzed using thematic analysis following a deductive-inductive approach.

**Results:**

According to study participants, financial burdens, challenges in familial dynamics, mental health issues and aging population difficulties were common social problems in Saudi primary health care. Action on SDH in primary care was hindered by 1) lack of physician knowledge or training; 2) organizational barriers including time constraints, patient referral/follow up; 3) patient cultural norms and 4) lack of awareness of physician’s role in managing SDH. Enablers to more socially accountable care suggested by participants includes: 1) more education and training on addressing SDH in clinical care; 2) organizational innovations to streamline identification of SDH during patient encounters (e.g. case finding questionnaire completed in waiting room); 3) better interprofessional coordination and clarification of roles (e.g. when to refer to social work, what support is provided by physicians); 4) identifying opportunities for broader advocacy to improve living conditions for marginalized groups.

**Conclusion:**

Enabling more socially accountable care requires a multipronged approach including leadership from the Ministry of Health, hospital administrations and medical schools. In particular, there is a need for: 1) training physicians to help patients in navigating social challenges; 2) improving clinical/administrative interprofessional teams, 3) mobilizing local communities in addressing social challenges; and 4) advocating for intersectoral action to prevent health inequities before they become more complex issues presenting to clinical care.

## Background

In recent decades, the relationship between primary health care (PHC) and the social determinants of health (SDH) has garnered increasing attention [1, 2]. Although frontline health workers continue to witness the detrimental impacts of social challenges on their patients’ health, a growing movement is advocating for increased social accountability in primary health care, and investments in clinical competencies to act on SDH [1, 3].

According to the World Health Organization (WHO), social determinants of health (SDH) are defined as “the conditions in which people are born, grow, live, work and age; these circumstances are shaped by the distribution of money, power and resources at global, national and local levels” [2, 4].

Gradually incorporating SDH into the PHC discourse reflects acceptance of the idea that social factors influence a patient’s presentation to his or her primary care physician and the ability to support patients in navigating these challenges can promote improved patient outcomes [1, 5, 6].

In 2008, the World Health Organization (WHO) released a report entitled “*Primary Health Care – Now More Than Ever*” that emphasized the importance of going back to the Alma Ata declaration’s definition of primary health care [7, 8]. The report urges adoption of a more socially accountable model of health care by taking increased action across a range of SDH and highlights the importance of action at the primary level of entry to care [8]. Doing so will lead to a better response to people’s needs and improve community health outcomes [1, 2].

Health systems are often slow to adopt such changes [9–11]. Recurrent visits to primary care clinics suggest that the underlying social causes of disease were frequently not addressed, and patients remain in the same social situations, living conditions, and unhealthy environments [9]. Some physicians consider issues such as domestic violence, poverty, and unemployment to be beyond their scope [9]. Other physicians report being untrained or unqualified to address their patients’ social causes of poor health [9].

Addressing patients’ social determinants of health is still in the process of being meaningfully embraced in the Middle East [12] and only a small number of articles published in the Eastern Mediterranean region call for adoption of a biopsychosocial approach in medical education and promoting the role of health workers in addressing the SDH more broadly [13–17]. The Saudi health care system has become one of the most advanced in the Middle East with a strong focus on health promotion [18, 19]. Nevertheless, literature on the integration of SDH in the Saudi primary health care setting remains sparse and little is published on levers for addressing SDH in clinical practice and improving support for marginalized patients [20].

.Marginalization is “a process through which certain population groups experience multiple social determinants concurrently. Thus, limiting their access to health promoting resources, while increasing their risk for poor health” [21]. An individual’s social position (gender, sexual orientation, race and ethnicity), their social environment and the resources available to prevent or fight disease; education, income and quality of their residential housing, all together produce an individual’s health. These complex interactions are the link between marginalization and SDH [21]. It is therefore our aim to report qualitative findings from a mixed methods study in Riyadh, that addresses this gap. Our study aims to explore the views of primary health care physicians in Saudi Arabia about addressing SDH in clinical practice, describe their current culture of practice regarding SDH and identify perceived barriers and enablers in asking and managing patient social challenges in clinical care.

## Conceptual Framework

To guide our empirical understanding of social determinants of health in Saudi Arabia and their effects on population health, we adopted the WHO’s conceptual framework for action on SDH, which takes note of the specific theories on the social production of health and disease [22]. This framework categorizes the SDH into three distinctive but interlinked classes: (1) structural determinants or social determinants of health inequities, (2) intermediary determinants, and (3) cross-cutting determinants such as social cohesion and social capital [22]. The conceptualization of marginalization within the SDH framework allows us to appreciate not only indviduals vulnerabilities but their resilience as well, thus allowing us to explore their experiences in how they cope with stress and adapt to social change [21, 22].

## Methods

### Study design

We used a qualitative descriptive approach within a naturalistic inquiry paradigm to explore the complex phenomenon of the barriers and enablers to addressing SDH in clinical care [23]. In depth interviews were used to explore Saudi primary health care physicians’ perspectives on caring for underserved and marginalized patients which is crucial in understanding the local setting and Saudi primary health care context [24]. In addition, triangulation with a focus group involving clinical social workers based at the same clinical site provided a nuanced and multi-faceted understanding of how social issues are addressed in the primary health care space. While physicians provided immediate clinical care in a primary health care practice that often includes discovery of SDH, it still requires further exploration and management, and this unique care is provided by a clinical social worker. Exploring a culturally homogenous group such as social workers at the same teaching hospital allows a more open dialogue about shared work experiences, content information about the local referral organizations, and suggestions for improvement on service delivery for vulnerable patients thus reflecting the social realities and knowledge [25, 26].

#### Setting

The kingdom of Saudi Arabia is the second largest Muslim country in the Arabian Peninsula, with its current population of 35.5 million people [27, 28]. Riyadh is the capital and largest city in the kingdom with a population of 6 million people [28]. The current median age in Saudi Arabia is 27.5 years of age with a full life expectancy of 75.5 years of age [28]. The country’s largest economic asset is oil production and trade [29]. Saudi Arabia finds itself in a highly transitional period in terms of social reform and economic reevaluation [29]. Saudi Arabia’s culture is a mix of Arab traditions and customs with an Islamic worldview [24, 29]. The Shariah law governs life such as politics, economics, finances, family, hygiene, and social issues [14, 19, 24, 29]. Family is a vital part of Saudi society, a an individual commonly has an extended support system that includes parents, grandparents, siblings, aunts, uncles and cousins [29, 30]. Extended family ties are strongly encouraged and maintained. Family is considered an essential part of an individual’s identity [14, 19, 24, 29, 30].

This study was conducted at King Khaled University Hospital (KKUH) in Riyadh, Saudi Arabia, a public teaching hospital that provides primary and secondary care to low- and middle-income patients in the Northern part of the city. It includes a large family medicine unit with key academic collaborators in the Department of Family and Community Medicine.

#### Sampling and recruitment

##### In-depth interviews with primary health care physicians

A maximum variation purposive sampling technique was used to select the 17 physician participants with different duration of work experience (i.e. 5 with less than 10-year experience, 5 with 10-15 years of experience, and 7 with over 15-year experience), gender (i.e. 10 male, 7 female) and nationality (i.e. 12 Saudi, 7 non-Saudi) to obtain a wide range of viewpoints [23]. Primary health care physicians were recruited via email invitations that explained the study’s purpose and what participation in the study entails. A mobile text invite was also sent to all primary health care physicians working in the Department of Family and Community Medicine at KKUH. Active recruitment stopped when data saturation was reached, where “no new information or themes are observed in the data” [31] in the total 17 interviews [23].

Prior to commencing each interview, an informed consent and permission to audio-record from the participant was discussed and signed. Interviews were conducted in both English and Arabic, based on the participants preference, and they each lasted on average 30-40 min.

##### Focus group with social workers

As part of the multistage qualitative data collection process, a follow-on focus group was conducted with 4 clinical social workers working at KKUH primary health care clinics. This allowed a more in-depth understanding from an additional viewpoint of the dynamics within the same clinical practice environment. A maximum variation purposive sampling technique was similarly used for the focus group to identify a diverse group of clinical social workers to obtain a wide range of viewpoints [32]. An email invitation letter was sent which includes information about the study’s purpose to the head of the Department of Clinical Social Work at KKUH. Active recruitment stopped when data saturation was reached [26].

#### Data collection and analysis

Data was collected using semi-structured interview guides with open-ended questions informed by the Theory of Planned Behavior [23, 33]. The Theory of Planned Behavior is a widely used framework to help increase clinician uptake of evidence-based practices. This theory is broken down into psychological/behavioral constructs about what guides human behavior [33], suggesting that attitudes towards the behavior, subjective norms, and perceived behavioral control influence behavioral intention to change and that the latter is a strong predictor of future behavior [33]. Interviews explored perceived barriers and facilitators to taking action within established norms of Saudi clinical practice, and knowledge about the available local resources and support organizations for these patients. The interview guide was piloted with two local clinician-researchers and the content was adjusted prior to using the guide in the study [34].

All interview and focus group recordings were transcribed and translated verbatim from Arabic to English. A second translator was recruited to review the transcripts for accuracy. Transcripts were then analyzed using a thematic content analysis approach as described by Crabtree and Miller [35, 36]. A pre-established deductive coding frame was used as well as an inductive approach to categorize and identify emerging themes [35]. The deductive frame centered around five predetermined questions: 1) the most common social challenges faced by patients presenting to Saudi primary health care clinics, 2) approaches to asking about social challenges in a sensitive way, 3) the perceived barriers to addressing social challenges in a clinical setting, and 4) opportunities and enablers that can be used to overcome these barriers and promote more socially accountable care.

Textual data were coded to categorize and identify emerging themes that were then grouped on the basis of similarities, differences, and participants’ key phrases [35]. Data coding was done by two independent researchers (BA and AA), the codes and categories identified by the two were compared and any disagreement was resolved. The data analysis process was done manually without the use of analysis software and lasted about 3 months. Further analysis techniques included: coding the whole corpus of text, reassessing the consistency of the coding, and finally making sense of the themes or categories identified. This helped improve relevance while helping understand the social realities of Saudi health care [37]. To ensure rigor, it was important to illustrate the richness of the data and convey it to the reader by an explicit representation of the congruence between the themes identified and the statements made by the participants [38], we used the following four criteria (credibility, dependability, confirmability, transferability) to establish trustworthiness in this study [38, 39].

## Results

Study findings are presented in terms of four key themes emerging from analysis: 1) kinds of challenges faced by marginalized patients presenting to primary care; 2) physician approaches to addressing social challenges; 3) barriers to taking action on SDH, and 4) opportunities for promoting social accountability in clinical care.

### Types of marginalized patients presenting to primary care

The most common social challenges among patients presenting to Saudi primary care clinics were related to financial constraints, family dynamics, mental health challenges and difficulties related to old age. As described by a Saudi male consultant in family medicine with 30 years of experience:

“What makes my patients vulnerable in our clinic is multifactorial. Some of them are vulnerable because of social disadvantages which include poverty, low income, low social support from the family or from the community in general. Other than social, it could be psychological: many have depression or anxiety which sometimes leads to more severe disability.”

In terms of patients’ *financial issues*, participants discussed low-income including unemployment affecting patients’ ability to afford medication and access means of transportation to attend appointments at their clinic. In her 10 years of practice, a Saudi female family consultant said: “financial difficulties are unfortunately the major social cause of poor health among my patients,” and particularly for women. A Saudi female family consultant and assistant professor who trains and teaches residents and medical students shared an example:

“A patient who is a widow … (who) is responsible for her children and grandchildren…starts sobbing during the consultation, and you find out her financial status is so awful that she sells her own stuff to spend on her kids.”

She explained that these stressors can manifest as somatic symptoms that can be misdiagnosed as physical illnesses, describing a patient who often complained of tension headaches and Irritable Bowel Syndrome symptoms.

With respect to *family dynamics*, patients may face domestic abuse or violence, marital dispute and divorce, spousal neglect, and lack of family support. A Saudi female family consultant and assistant professor, who has been working for KKUH for almost 4 years gives an example;

“The other common problem is family abuse. I can tell you hundreds of stories about it, and the most recent is a widow who had a son and four daughters who were abusing her. They tried to kick her out of the house and threatened her.”

Regarding *mental health issues*, participants said that patients, in particular those who don’t realize they may be suffering from depression and anxiety, present with a somatization disorder and with psychosomatic complaints. Sometimes these mental health issues stem from a specific situation the patient is experiencing and are consequences of the financial and family dynamic challenges as the following quotations illustrate;

“I once had a schoolteacher who kept coming with symptoms of depression and low mood such as headaches, fatigue, and insomnia. I found out later on that she was a second wife and had to take care of her husband's children from his first in addition to her hectic work schedule.” F_Saudi_17/7_003

Finally, regarding *old age difficulties*, participants described their elderly patients as often having common chronic diseases such as diabetes and hypertension, however it was not the main challenge these patients were having. Many respondents described the impact of social isolation on the elderly and the compounded health-related consequences due to their loneliness. For example, one male doctor described a patient who is “lonely to the extent that he comes to the hospital by himself in a taxi because none of his sons were available to bring him to the appointment.” M_Saudi_28/6_001.

### Physician’s approaches to asking about social challenges

The majority of study respondents claimed that understanding their patient’s social history is part of their role as primary health care practitioners and family consultants, and an essential aspect of effective treatment: “I ask them directly...we need to ask, we have to solve the case, the puzzle. Because we have to find out if it’s really an organic problem or something else.” M_Saudi_23/6_007. A similar sentiment was expressed by a female doctor who saw it a necessary part of patient history taking: “I honestly ask all of the patients as soon as I have doubts… Even if it takes an hour, because you might be really saving him or her in this session... so, it is essential to ask, not a duty. It is something beyond duty to ask.” F_Saudi_17/7_001.

Several respondents provided insight regarding how to initiate these conversations such as asking patients for permission to broach a topic: “I need to ask you about a sensitive topic, would you mind that? If you don’t mind, we need to get into your situation at home.” M_NonSaudi_28/6_002. A direct approach was also described:

“Sometimes patients want you to ask, so I ask…. How do I ask? Directly to the bottom line! Aunt, how is your financial condition? How is your relationship with your husband? Are you children treating you well? In a nice way using colloquial language, I try to use some words from their dialect. Or try to be close to her understanding and make the questions more friendly.” F_Saudi_17/7_001

While aware of the importance of asking the patients about their social challenges, a minority of respondents expressed hesitancy in intervening on these issues: “I learned the hard way when I moved here not to intervene in these sensitive issues... If you sense something is wrong, you have to ask, but here the society and community is really reserved...It’s better off not to ask in the first place.” F_NonSaudi_17/7_004.

### Physician’s perceived barriers to taking action on SDH and socially accountable care

Although primary care physicians in Saudi report caring for a wide range of patients experiencing various forms of social adversity, they report many barriers to asking about and addressing these challenges in clinical practice. These barriers were categorized starting with factors related to both the physician and patient, up to a broader community-societal level (see Table 1).

**Table 1:** Saudi physician’s perceived barriers and enablers to socially accountable care

**Physician’s perceived barriers to socially accountable care**	
***1. Micro-level*** **physician- patient factors** -Biomedical bias -Lack of physician knowledge or training -Patient’s refusal, cultural beliefs and expectations ***2. Meso- level*** **organizational factors and Interprofessional relationships** -Clinic structure -Time constraints and difficulties to follow up - Disconnect between primary care physicians and social workers ***3. Macro-level*** **societal factors** -Disconnect between the primary care clinic and the outside community hinders referral of patients to local support agencies	
**Physician’s perceived enablers to socially accountable care**	
***1. Micro-level*** **opportunities for promoting social care** -Graduate & postgraduate education/training -Physician workshops & seminars -Educating patient communities on SDH ***2. Meso-level*** **opportunities for promoting social care** -Use of nurses for screening & triaging patients to minimize consultation time -Foster stronger integration between PHCs and social work departments -Multidisciplinary primary care team with a clear referral strategy ***3. Macro- Level*** **opportunities for promoting social care** -Identify & publicly advertise local resources and organizations for patients’ social aid and support	

#### Micro-level physician-patient factors

A number of micro-level factor hindered efforts to address SDH in the clinical encounter. Among these was a prevailing biomedical bias in the medical profession. While some participants perceived the field of family medicine and primary care as being holistic and requiring a biopsychosocial approach other were disinclined to take action on SDH during their clinical encounter with a patient. A young male Saudi family physician explained why some of his peers feel reluctance:

“They don't want to hold the responsibility, but rather refer to the social worker or the police... some are refusing to take the case, and make the real job, the job a primary care physician should do.” M_Saudi_23/6_007

Lack of physician knowledge and training on how to address SDH was another factor hindering action. Participants felt they didn’t have enough information and guidance on how to address their patient’s social challenges. As one female practitioner remarked: “Unfortunately, due to our lack of knowledge in this area, we tend to focus more on the physical and organic symptoms and neglect the underlying social issues.” F_Saudi_23/6_003.

Most participants were not adequately educated on the role of a social worker within their clinical setting and were not aware of the diverse set of services, resources and local support organizations they are capable of providing for the patients: “I don’t know what the social office actually does. Other than giving financial support, we don’t know what they offer our patients.” M_NonSaudi_28/6_002.

Finally, patient-related barriers include patients’ refusal to answer questions regarding their social challenges or to allow physicians to take action. As explained by a female Saudi primary care physician: “The patient refuses most of the time to acknowledge the fact that their medical symptoms might be stemming from non-medical and non-organic reasons.” F_Saudi_23/6_003.

Patients’ reluctance to openly discuss their social struggles was explained by their lack of knowledge about the scope of care a family doctor is capable of providing. As the same physician explained: “The people here in our community are used to -- when they go to the primary care centers-- the doctors just listen to the complaint and they immediately prescribe medication.” F_Saudi_23/6_003.

Patients’ resistance to answer questions or allow physicians to take action are due to several factors including fear of being exposed to other family members, and community stigma. To illustrate, one participant said: “Especially in our community, patients are really shy about these issues…There’s a lot of resistance due to the stigma and fear of being labelled as mentally ill and getting addicted to antidepressants.” F_Saudi_23/06_003.

Despite the large number of domestic abuse cases seen at the primary care clinics, and reticence in discussing these issues openly, physicians are often faced with situations of patient hesitancy. A Saudi female physician explained that many female patients refuse to get referred to official bodies of authority handling family violence. As another female doctor elaborated: “There is a lot of resistance from the patient. Most of the time it is out of fear of the husband and fear of getting divorced and the husband taking custody of the kids by law.”

A physician’s inability to communicate with patients and understand their concerns was a common barrier reported by participants. Information can be disregarded, lost or misunderstood in the consultation due to different cultural backgrounds, languages or dialects. One Saudi male physician who worked in three different regions explained: “The new foreign doctors have to adapt to our community, so there is a lot of misinterpretation and a lot of communication errors (….) Even though you’re a Saudi and working in a Saudi city, if I went to one of the villages of the south region or south west, I’d be totally lost because they have their own type of phrases.” M_Saudi_23/6_007.

#### Meso- level organizational factors and Interprofessional relationships

A second category of barriers were management-related such as time constrains, difficulties with patient follow up and disconnect between the primary care department and the social workers.

Regarding clinic time constraints**,** participants expressed that given their caseload and frequent overscheduling, appointments were not long enough to adequately address the SDH a patient may be facing: “For a single doctor at the clinic, it is too much... and we cannot do this for everybody.” Poor patient follow-up is also a constraint. For example, the KKUH appointment system is organized such a way that patients may not always see the same physician. This hinders continuity of care and follow up which are essential to wholistic care.

Lack of communication between social work and primary care departments at the hospital was also reported as hindering SDH referral. One female Saudi family physician with 7 years of experience described “… miscommunication and disconnect between our department here and the social workers” (F_Saudi_23/6_003), while her counterpart social worker confirmed her account…” (there is) no connection with the social worker. Our connection is just with paper, unfortunately.” (M_Saudi_26/6_005). Social workers were also concerned about the division of tasks between physicians and themselves, including role boundaries, as one of the female social workers expressed here “You are asking the doctor to play two roles here. That will require so much time in his clinic.” F_Saudi_SW_001.

#### Macro-level societal factors

A third category of factors hindering the referral of patients with SDH issues concerned the disconnect between the primary care clinic and community-based support agencies and other resources. Although some participants only knew about a few local support agencies and organizations from personal encounters or advertisements, the majority of them did not. Those who did know about these resources continued to mention how difficult it was to reach them and have direct access when needed. This was a common finding expressed by participants across all demographics as stated; “I don’t know about any organization outside the hospital that I can refer my patients to.” M_Saudi_26/6_005.

### Opportunities for promoting social accountability in clinical care

A number of recommendations to help overcome barriers in managing SDH among socially marginalized patient populations were suggested by participants.

#### Micro-level opportunities for promoting social care

A key entry point for promoting social care through graduate and postgraduate education and training. A Saudi assistant professor and family medicine consultant emphasized the importance of including a chapter in the medical school curriculum at all levels that specifies the relationship between SDH and poor health. “The curriculum has no information about the social aspect or social work in detail, it just briefly mentions the biopsychosocial history taking” F_Saudi_26/6_006.

Physician workshops and seminars were also suggested. Developed with the guidance of social workers, these events would be designed to train physicians on how to better address patients with social challenges, the standardized hospital process and steps needed to refer and support these patients, in addition to introducing the physicians to the various local resources and organizations available. Physicians are motivated to learn as illustrated by the following quote:

“I would love to attend some sort of physician workshop or even have a presentation about this issue and the available organizations” (F_NonSaudi_17/7_004), “We, as doctors, need to educate and accustom ourselves. Even if nobody shared the information [to follow-up regarding a referred patient], we are supposed to take the first step and go and ask the social workers ourselves.” F_Saudi_17/7_001

It was further recommended that education about SDH be extended to patients. In addition to increasing patient awareness about the scope of family medicine and primary health care services, physicians should stress to patients that opening up and disclosing information regarding one’s social challenges, is integral part of treatment: “I think we need to educate the patients that non-clinical data is very important.” M_Saudi_26/6_005.

#### Meso-level opportunities for promoting social care

At the organizational level, recommendations included nurses for screening & triaging patients to allow for longer appointments when necessary: “a screening service for social problems, and some sort of a triage for different types of patients.” (F_Saudi_17/7_003) could be implemented.

Improving the communication between the primary health care and the social work departments was also considered integral to creating a stronger, more functional bond that would be beneficial for both departments and, ultimately, for patient care and support. Suggestions included greater collaboration by dedicating a specific social worker to handle primary health care department patients or by localizing SW offices in close proximity to the PHC department. One primary care clinician noted that having a dedicated social worker for each clinic would be ideal, while another suggested: “… if they had an office next to our clinic...you save time for both the doctor and social workers because the help is a teamwork, which is what a primary care clinic should be, to support each other for the benefit of the patient.” M_Saudi_23/6_001.

Key to effective SDH care is a multidisciplinary primary care team with a clear referral strategy. Included on this interprofessional team is a dedicated social worker, thus fostering greater integration between clinical and social work departments and enabling a well-articulated referral system that allows for direct communication, feedback and follow-up between social worker, clinician and patient. As part of this referral process, it is critical that the physician understand “what the social worker is going to do with the patient, so it can be explained ahead to the patients.” F_Saudi_17/7_002.

#### Macro- level opportunities for promoting social care

In terms of macro-level opportunities for promoting social care, a central action is to identify and publicly advertise local resources and organizations for patients’ social aid and support. Public awareness of available services, local organizations and support agencies should be displayed in hospital hallways, waiting areas and even shopping malls to “stimulate interest in people” F_Saudi_17/7_001.

## Discussion

Although the term “Social Determinants of Health” was unfamiliar to many of the primary care providers in the study, it was generally understood that to effectively identify and address a patient’s health needs, it is often necessary to” dig in their social history” and in some cases, uncover their “hidden agenda”. This stance towards SDH in clinical care corresponds well with findings from a Canadian study involving physicians who had previously practiced in the Middle East [40]. While a conceptual understanding of being a socially accountable health practitioner exists in the Arab world, the construct of SDH is relatively new. This could explain why many publications in Gulf Cooperation Council (GCC) countries focus on specific areas of social disadvantage (e.g., violence or child abuse), versus a wholistic social determinants approach [13–17, 40]. This could be related first component of the WHO’s conceptual framework for action on SDH, the socioeconomic position of the patient as a structural social determinant of health [22].

Like other studies in the Middle East [40], our research found that in Saudi primary care clinics, physicians often see many patients struggling with issues relating to poverty, exposure to family violence, mental health challenges and frailty in old age. Forms of marginalization found in the Saudi patient population presenting to primary care are comparable to those seen in a Canadian Family Medicine center in Montreal serving a large population of immigrants and refugees [41]. Among these were patients with mental health problems, people living in poverty, single parents, substance abuse, isolated seniors and victims of abuse and neglect [41]. These findings elude to social vulnerability possibly being a global issue that may require a universal framework of action focused on the social determinants of health such as the WHO’s conceptual framework for action on SDH to deepen our understanding of the type of change needed to address these challenges [21, 22].

A first step in taking action on the social determinants of health is to “ask” about them. However, inquiries about a patient’s social struggles and life challenges requires a relationship between provider and patient that is built on trust. Primary health care practitioners in our study perceived that asking questions about a patient’s social history is an integral part of their role. Some were confident enough to ask directly or probe indirectly, while others found it difficult and challenging, fearful of being intrusive and offending their patients. Several barriers were noted that align with the social accountability framework [5, 6, 42]. Starting from the micro or individual level, many physicians considered issues such as marital problems, domestic violence, poverty, and unemployment to be beyond the scope of the biomedical model in which they have been trained. At the meso level of community level, social issues are not always acknowledged or discussed due to sociocultural barriers, further complicating the process of providing care. Finally, at the macro level, inadequate advocacy for or policy attention to the social determinants of health are apparent. Clearly, further efforts at the meso and macro levels are necessary to support physicians at the micro level.

Physicians also reported being untrained or unqualified to address their patients’ social causes of poor health, emphasizing gaps in knowledge including understanding the role of the social worker on site in their hospital, being familiar with available resources, and knowing which organizations and support agencies to refer patients to. This is consistent with the current literature and the hurdles faced were similar to those encountered by western physicians, who also lacked the appropriate training to deal with SDH [15, 40, 41]. There is also fear of retribution for getting involved in sensitive areas. In a study in Bahrain, hesitation in reporting child abuse in clinical practice, was mainly attributed to a desire to avoid conflict with the family and lack of knowledge about legal reporting mechanisms [43].

Hesitation, however, was not confined to healthcare providers. Several physicians’ described scenarios in which patients refused to answer sensitive questions or failed to connect their social and health concerns. For example, a patients’ level of education and understanding of how social stressors can lead to poor health might delay their visit to a primary care physician, resist questions relating to their social struggles or refuse to accept help even if they disclosed. Another factor influencing patients’ decisions about their health is stigma, and fear of being exposed as someone needed psychosocial or other forms of social or economic assistance. This is supported by the work of Hofstede on collectivist cultures [44], in which individuals give precedence to the welfare of the group rather than the individual. Against this backdrop, patients may not want to bring harm to their families, even if the price is their own wellbeing.

Beyond issues related to the awareness, comfort and competency of health workers to address SDH, there are organizational and interprofessional barriers at the meso-level. Organizational barriers include those related to clinic processes and structures i.e. consultation time constraints, clinical workload and difficulty following up with patients. For example, a demanding workload influences how physicians perceive SDH and whether they have the time and energy to address them. The development of policies and procedures, the work life balance for practitioners is necessary. The lack of policies contributes to the structural determinants of health as described in the WHO’s conceptual framework for action on SDH [22]. This is also issue in the western contexts, where the most common reported barriers to addressing SDH included time constraints, knowledge deficiencies, lack of skills and training pertinent to socially sensitive situations, and deficits in evidence-based research on useful interventions or tools on SDH what are tailored to the needs of practitioners [45]. In 2011, in an online survey, 87% of American physicians reported being aware that social factors impact health, yet only 20% felt comfortable discussing these issues with their patients, citing heavy turnover of patients and lack of manpower [46].

The disconnect between social workers and primary care physicians is an example of interprofessional barriers to addressing SDH at the meso level. Barriers perceived by social workers mainly related to a lack of communication and collaboration with physicians with respect to patient management. Social workers felt that lack of knowledge about their role and scope of services delayed the required referral process to their department. However, less clear is the division of tasks between both departments. Social workers believed patients were more comfortable disclosing sensitive matters away from the clinic. From the physician’s perspective, close proximity between the primary care clinics and the social workers office was seen as desirable but potentially threatening to social workers who may fear role encroachment [47]. Of note, while the hospital where the study was conducted (KKUH) has a social worker on staff, the majority of primary care clinics and centers belonging to the Ministry of Health do not. Therefore, it is important to train doctors to address social determinants of health in the absence of social workers.

Finally, at the macro-level, societal factors hindering primary care physician’s action on SDH in clinical care are related to the disconnect between clinic and community. An often-repeated barrier by participants in the study was a lack of knowledge about available resources for vulnerable and marginalized patients, how they might be accessed and once accessed, how to ensure that appropriate help and support are provided.

Suggestions for improvement and opportunities to enable physicians to more effectively address SDH align with the social accountability framework and must address micro, meso and macro levels of change [5, 6, 42, 48]. At the micro level, recommendations include increasing education about SDH starting with curriculum for medical students and providing graduate and postgraduate training on the actions and approaches for dealing with SDH in clinical care. Workshops and presentations for physicians and consultants on new developments in SDH approaches and their efficiency in clinical care are also recommended.

Family medicine and primary health care physicians could also benefit from training on Trauma-informed care which emphasizes the relationship between physician and patient as a key component of care and strengthens their connection to their patient. The Trauma-informed care approach guides physicians who might be unsure of how to approach some difficult and sensitive topics that are central to a person’s health. Physicians are susceptible to society’s collective denial of child abuse and neglect. Physicians are morally called on to provide compassionate and healing care to the survivors of such trauma. Given that adverse childhood experiences (ACEs) can be a root cause of many illnesses clinicians encounter on a daily basis, trauma-informed care can improve patient healing and outcomes, and lead to greater professional satisfaction [49]. This approach can be further supported by educating patient communities on SDH and how doctors can address these issues within their clinical practice.

At the meso-level of action, one recommendation from primary health care physicians was greater reliance on nurses for screening and triaging patients, with the goal of freeing up consultation time for SDH assessment and referral [50]. This would also serve to strengthen the connection between primary care physicians and social workers, contribute towards the development of a socially accountable, productive and multidisciplinary team of health workers capable of enacting effective SDH referrals.

At the macro-level of action, participants stressed the need for better media coverage and campaigns to identify available resources, support organizations and local agencies for addressing all kinds of social determinants of health. This effort could be supported by ongoing efforts of the Ministry of Health and Ministry of Human Resources and Social Development to implement the Saudi government’s new direction and vision towards a more efficient and accessible primary health care network.

### Study limitations

A major limitation of the study was its predominant focus on government teaching hospitals, and medical doctors and the implications of both for the transferability of research findings. Other non-academic hospitals also run by the Ministry of Health serve more diverse and socially vulnerable populations but were not included in the study as they lacked social worker services. A related limitation was difficulty recruiting social workers into the study, with participation limited to a 4-person focus group of social workers. Nevertheless, valuable insights were produced regarding shared work experiences, local referral organizations, and suggestions for improvement on service delivery for vulnerable patients [49]. In addition, the study sample did not include the perspectives of patients themselves, which would offer an important opportunity to further triangulate findings about the value integrating a social determinants approach. Finally, valuable insight would have been gained had we focused on how the participants spoke about their experiences in addition to what they said. Doing so would deepen our understanding of our participants’ experiences and help us avoid reproducing marginalization.

## Conclusions

Results from this study emphasize an important new direction for primary health care research and advocacy in Saudi Arabia. In particular, they provide fresh perspectives on how primary health care physicians can more effectively address SDH within the clinical setting, and beyond as advocates for system and policy change that recognizes SDH and supports linkages between health services and community-based organizations. Further, critical knowledge gaps are identified that will guide health education policy and the scale-up of social accountability approaches to undergraduate, post-graduate, and continuing medical education in Saudi Arabia and throughout the Middle East.

Study results also underline the need for greater interprofessional cooperation between physician and clinical social workers, who are trained to address the social challenges of their patients and to provide them with the support they need. Social workers have a wealth of knowledge and experience in availing support resources in the community and are well placed to assist family physicians in taking action on social determinants. At the same time, sensitivity to role encroachment, and conflict minimization should be exercised.

Finally, primary care physicians in Saudi Arabia understand the importance of addressing their patient’s social causes of poor health but require an enabling policy environment that values a social determinants approach and supports them in overcoming barriers in routine clinical practice. In this regard, investments in operationalizing a social accountability approach are needed which are compatible with the government’s commitment to achieve sustainable development goals (SDGs) focused on the social determinants of health. The effectiveness of primary healthcare systems will be enhanced if they support partnership between primary care physicians and community-based services given that so much of health lies beyond medical care.

## Data Availability

The data from this study will not be made publicly available as it contains personal identifiable data.

## Abbreviations

SDH: Social Determinants of Health
KKUH: King Khaled University Hospital
PHC: Primary Health Care
WHO: World Health Organization
GCC: Gulf Cooperation Council
ACEs: Adverse Childhood Experiences
SDGs: Sustainable Development Goals
